# Why do scientists disagree? Explaining and improving measures of the perceived causes of scientific disputes

**DOI:** 10.1371/journal.pone.0211269

**Published:** 2019-02-07

**Authors:** Nathan F. Dieckmann, Branden B. Johnson

**Affiliations:** 1 Oregon Health & Science University, Portland, Oregon, United States of America; 2 Decision Research, Eugene, Oregon, United States of America; Saint Peter's University, UNITED STATES

## Abstract

There has been increasing attention to understanding how laypeople explain disagreements among scientists. In this article, we evaluate the factorial validity and scale/item functioning of a Science Dispute Reasons scale (Study 1) and test specific hypotheses about demographic, individual difference, and topic-related variables that may explain why some reasons are perceived to be more likely than others (Study 2). The final scale included 17 items grouped into three reason factors (Process/Competence, Interests/Values, and Complexity/Uncertainty), which is largely consistent with previous research. We find a mixed pattern of global and specific impacts on reason likelihood ratings from a range of variables including political ideology and conspiracist ideation (primary mediated through perceived credibility of science), science knowledge, and topic-related variables such as knowledge of and care about the dispute in question. Overall, science dispute reasons appear to be more strongly driven by attitudes and worldviews as opposed to objective knowledge and skills. These findings represent progress in understanding lay perceptions of the causes of scientific disputes, although much work remains. We discuss the implications of this work and directions for future research.

## Introduction

There has been increasing attention to understanding how laypeople explain disagreements among scientists. These efforts have been driven by concerns that lay perceptions of scientific disputes might undermine scientific credibility, furthering misunderstanding of how science operates and/or leading people to ignore scientific advice (e.g., [1, 2, 3, 4]). As we detail below, the literature so far has not yet converged on the reasons offered by laypeople for intra-science disputes, and even less on which competencies, beliefs, attitudes and demographic factors are associated with favoring one reason over another.

In an attempt to provide more structure for this continuing question, we report the results from two studies. The first major contribution of this work was to produce a psychometrically reliable measure of Science Dispute Reasons for use with US samples. The second major contribution was to test a wide range of potential individual-difference correlates of lay reason perceptions across diverse dispute topics. Such work is needed to understand how lay reasons for scientific disputes might converge or diverge across situations or individuals. We find that several factors including science knowledge, perceived credibility of science, conspiracist ideation, and political ideology predict Science Dispute Reasons depending on the specific scientific dispute in question.

## Background

### The causes of expert disagreement

Why do scientists disagree in the first place? One set of potential causes focuses on the experts themselves. One or more of the experts may be making an inaccurate claim because of incompetence (i.e., they are not experts at all [5]) and/or the fundamental limits of human judgment [6], or they may be intentionally or unintentionally biasing claims because of idiosyncratic attitudes, beliefs, or personal interests [7]. Another expert-focused cause might be different methodological choices that stem from individual scientists’ skills or preferences, or from historical developments in their respective fields or sub-disciplines. Alternatively, disagreements among experts within scientific fields may be due to irreducible uncertainty of the world itself and could be conceived of as a part of the normal process of science [8, 9]. From this perspective, it is inevitable that experts will disagree when confronting complex and uncertain real-world problems. It is the complexity and inherent uncertainty of the world that leads to disagreements about how to conceptualize problems, the research methods that should be used, etc. From a conceptual standpoint, these various expert- and world-focused reasons are neither logically nor practically mutually exclusive. For any given dispute among scientists there might be multiple causes, and these causes might differ from one dispute to another. Our concern here, however, is with *lay perceptions* of the reasons or causes of such disputes.

### Measuring and predicting lay perceptions of expert disagreement

Several studies have explored reasons that laypeople might offer for why scientists have disagreements, and/or attempted to explain or predict why people might offer one reason rather than another. For example, educated Finns asked about food-additive disputes favored self-interest as a reason, while less educated Finns favored general difficulty in gaining scientific knowledge [10]. Dieckmann et al. [11] asked members of the U.S. public to rate a wide range of topics (seven general topics, e.g., terrorism; economics) on six different possible reasons for scientific disputes (complexity, randomness, lack of knowledge, incompetence, bias, and unwillingness to admit uncertainty). More educated or cognitively able participants thought scientific disputes were more often due to the topic’s irreducible complexity/uncertainty and to expert bias (e.g., personal values or external social forces influencing scientific claims), while the less educated or cognitively able favored scientists’ incompetence as a reason. Johnson and Dieckmann [12] examined reasons in another U.S. sample using specific scientific disputes related to dietary salt, dark matter, and nanotechnology. Reasons for science disputes were best described by a three-factor structure encompassing interest/values, process/competence, and complexity/uncertainty reasons. Reasons the authors thought conceptually distinct—i.e., scientific processes versus competence, and self-interests versus values/worldviews—were not seen as separate by lay respondents. Belief in scientific positivism and general perceptions about the credibility of science and scientists were the strongest predictors, but more in terms of how strongly reasons were rated as “likely” explanations of scientific disputes than for which reasons were favored. Neither education nor familiarity with scientific reasoning drove perceived reasons (cognitive ability was not examined in this study). Thomm et al. [13], using highly educated German subjects (88% with an academic degree in one study), found reasons for disputes over medical and climate change topics fell into four categories—complexity, researcher motivation (self-interest; values were not included), research process, and researcher competence—and produced a reliable Explaining Conflicting Scientific Claims (ECSC) scale. Process and complexity were favored reasons, with motivation following. These studies examined disputes between individuals, rather than groups of scientists as in previous studies. They found motivation and (for medicine, not climate change) competence reasons favored more when the disputants were an industrial and a university scientist. Explanatory factors probed were manipulated researcher affiliation, and prior knowledge and personal importance of the topic (nonsignificant overall).

More recently this research group [14] explored perceived dispute reasons in two domains (biology and history) in a sample of Israeli university students (*N* = 184). In the biology context, the favored reasons were topic complexity and research methods, whereas topic complexity and researcher motivations/background were the favored reasons in the history domain. They also found that epistemic perspectives—i.e., individuals’ assumptions about the nature of knowledge—were related to science dispute reasons (also see Discussion section). Based on a structure by Kuhn et al. [15], epistemic perspectives include absolutist (knowledge is objective so one expert must be correct), multiplist (knowledge is subjective and uncertain so we can’t say which expert is correct), and evaluativist (knowledge is subjective and uncertain but competing claims can still be judged to be more or less valid) forms. The absolutist perspective is equivalent to the scientific positivism belief we discuss below. A thorough discussion of these viewpoints and their implications is beyond the scope of this paper (see [14]).

Papers to date have differed in the number and type of topics they have used to illustrate intra-science disputes. They have also differed with respect to whether disputes were between individual or groups of scientists, their reasons measures, explanatory factors tested, and sample nationality. Laypeople might more easily attribute disputes between individual scientists to self-interest or values than for large groups of scientists, who may not share the same type of employer or motivations. Nationality might or might not affect results, depending upon whether there is a globally similar response—e.g., because the institution of science is done the same way everywhere and laypeople interpret science the same way wherever they live—or culturally variant responses. These differences in method make it difficult to evaluate the differences in findings. For example, clearly the *general* content of explanatory reasons laypeople offer for scientific disputes is similar. These include what scientists study (complexity), how they study it (methodological and other choices; competence), and their conscious or unconscious motivations for its study (personal interests, values). The limited number of studies to date in different countries also suggest some convergence on these categories of reasons by laypeople, implying that cultural differences might be minimal. However, this conclusion must be tentative because no study has yet applied the same measures to laypeople recruited from two or more cultures.

By contrast, despite this general convergence on the overall “reason” taxonomy the reasons’ priority vary more widely across these studies, as do the factors associated with these choices. The degree to which these differences reflect the different measures used is unclear. For example, we examined the English translation published of the items used in [13] and were concerned that the specific item content and phrasing, and rating instructions, might be problematic for use with the U.S. Public. Inspired by the Thomm et al. [13] and Johnson & Dieckmann [12] studies, the goals of Study 1 were to evaluate the factorial validity and scale/item functioning of a proposed scale measuring Science Dispute Reasons for future research with the U.S. public.

### Individual difference predictors and motivated cognition

A second goal, particularly for Study 2, was to probe factors associated with lay reason choices in more depth than ever before. Individuals’ perceptions of the most likely reasons for a given dispute are likely to be affected by both contextual and individual-difference factors. Context-specific factors include variables such as characteristics of the topic or domain in which the dispute occurs, any information provided about the disputing parties, or the content or basis for the dispute. The finding [14] of contextual differences between lay dispute reasons for biology and history might reflect, for example, perceived differences between these two disciplines specifically, or between natural sciences and humanities/social sciences. We explored context-specific factors in the present study only to the extent of varying the topic. However, we propose that the influence of individual-difference factors may function differently depending on the specific dispute in question. This is particularly true for ideology/worldview and conspiratorial thinking effects (discussed below), as these are activated to a lesser or greater extent depending on topic.

Relevant individual-difference factors include, but are not limited to, the following:

1) Cognitive/numerical skills, including measures of fluid intelligence and numeracy,

2) Science-specific knowledge, including science reasoning and science facts,

3) Science-specific beliefs including perceived credibility of science (generalized trust in science) and belief in scientific positivism (i.e., that the scientific method can be used to generate objective knowledge about the world), and

4) General attitudes/worldviews including political ideology (liberal-conservative) and conspiracist ideation (the extent to which people believe in conspiracy theories).

By including measures from all of these groups we could begin to address the general question of whether perceptions of science disputes are driven more by objective knowledge and skills (categories 1 and 2) or general attitudes and worldviews (categories 3 and 4). We expected that there would be a combination of global (affecting all dispute reasons) and specific (affecting only specific dispute reasons) associations between our individual-difference measures and Science Dispute Reasons. As an example of prior global associations, Johnson & Dieckmann [12] found that credibility of scientists (mistrust) and belief in positivism (examples of the science-specific beliefs of our taxonomy’s third category, above) were related to higher likelihood ratings for *all* Dispute Reasons. This suggests general attitude effects that are not Science Dispute Reason specific. With respect to perceptions of the credibility of science, this pattern of relations may be explained by some participants simply responding “highly likely” to any possible reason for disagreement because it is consistent with the belief that scientists/science are not to be trusted. The global positivism effect is somewhat more puzzling, however, and we sought to replicate that finding.

We also had several expectations related to specific Science Dispute Reasons—i.e., factors that alter the likelihood of lay selection of a specific reason but not others. We describe these roughly in the order of the four categories of explanatory factors outlined earlier.

First, with regard to cognitive/numerical skills and science-specific knowledge, we hypothesized (H1) that more science knowledge, and potentially higher cognitive ability, may relate to higher ratings of the complexity reason because these participants may be more aware of the impact of complexity and uncertainty on science practice. However, this effect may depend on the nature of the scientific claim under dispute. For instance, disputes will vary in complexity and perhaps those higher in science knowledge will assign higher likelihood ratings to complexity causes for objectively more complex dispute.

Second, general perceptions of the credibility of science might also have specific effects on dispute reasons. For example, individuals with less trust in science may be more likely to attribute science disputes to causes that are the “fault” of the scientists (e.g., personal interests). Below we also describe a potential mediator effect of such mistrust on reason choice.

**Fig 1:**
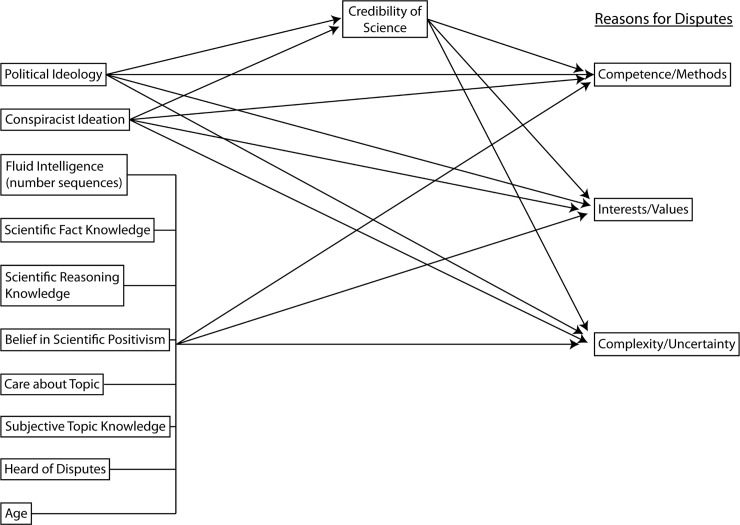
Study 2. Conceptual model tested for participants assigned to the salt and nanotechnology dispute scenarios.

Third, the last category in our taxonomy focuses on general attitudes/worldviews, which allow examination of the extent to which motivated reasoning processes are involved in perceptions of dispute reasons. Motivated reasoning or motivated evaluation describes the well-established phenomenon that people use existing mental models, beliefs and worldviews to guide information search, information interpretation, and judgment and decision making [16, 17, 18, 19, 20]. For example, political ideology, and the more complex but related variable of cultural worldviews, has been associated with trust and risk perceptions for specific science issues like climate change (e.g., [21, 22, 23], vaccines (e.g., [24]), and gun control [21, 25]. Political ideology has also been related to less favorable perceptions of the general credibility of science [26]. We hypothesize (H2) that political ideology will have direct and indirect effects on Science Dispute Reasons mediated through credibility of science (see Fig 1). These effects may be global (affect all Science Dispute Reasons) or specific to the more “damning” reasons of Interests/Values and Competence (being the fault of the scientist) rather than complexity/uncertainty (being caused by the world). Conspiracist ideation—a tendency to believe in conspiracies across a wide variety of issues—has also been related to science beliefs in several studies ([27, 28]; but see [29]). These effects have been found for particular conspiracy-conducive issues (e.g., conventional medicine, genetically modified food [26]) and to lower perceptions of the general credibility of science [26]. We hypothesize (H3) that Conspiracist Ideation will have direct and indirect effects on interests/values reasons specifically, given possible conspiracy theories built around outside interests and teams of scientists colluding to misrepresent scientific findings.

## Study 1

### Methods

#### Participants

All research presented in this paper was approved by the Decision Research Institutional Review Board (IRB). Members of the Survey Sampling International, Inc. online opt-in (i.e., non-representative) panel who were Americans 18 or older were randomly recruited June 28–29, 2017. This panel includes about 100,000 U.S. households who answer surveys for rewards. Our sample included 605 participants, with sample size targets set to exceed typical recommendations for IRT modeling and factor analysis (i.e., *n* ≥ 500; [30]). Respondents were 63% female (versus 51.4% in 2016 American Community Survey estimates for U.S. adults from the U.S. Census [31]), with a mean age of 54.1 years, range 18–88. Eighteen percent had a high school degree or less (40.5% for those 25+ in ACS estimates), and 52% (30.3% for those 25+ in ACS estimates) had a college degree or more. Some 83% identified as non-Hispanic white (73.3% in ACS estimates).

#### Item generation

We generated an initial set of 30 Science Dispute Reasons items based on the categorizations used for the prior Thomm et al. [13] and Johnson & Dieckmann [12] measures, but not including any of the actual items from these scales, nor identical instructions to those used earlier. Six items were included for each of five identified classes of reasons for scientific disputes: differences in research methods (e.g., in measuring tools, techniques for analyzing data, research designs, variables); differences in competence (e.g., different amounts of effort and care, credentials, experience), personal benefits (e.g., competition with other scientists, chance for money and promotion, employer persuasion), values (e.g., political beliefs, desire for elegant solutions, personal values), and thematic complexity/uncertainty (e.g., topic is too complex to get clear results, too many factors influence results for this topic). These categories echo the four categories of reasons proposed by Thomm et al. [13], and the five proposed—but empirically collapsed into three based on lay responses—by Johnson & Dieckmann [12], but the aim here was to produce clearer statements and instructions that might improve upon prior factoring with general population U.S. samples.

#### Procedure and measures

The survey instrument began with questions aimed at revealing prior awareness of and interest in disputes among scientists: “Sometimes a large group of scientists disagrees with another large group of scientists about the causes or effects of a natural event or technology. Have you ever heard about such a scientific dispute?,” and (for those who answered yes or don’t know) “Have you ever tried to decide which group of scientists was more likely to be correct?” For present purposes, we focused on the first answer (collapsing “no” and “don’t know” into a “never heard of disputes” category). Participants were then asked to read one of three scenarios about a real scientific dispute (Dark Matter, Marijuana, Sea Level Rise; see Appendix for wording), and answer questions on how much they cared about which position was correct (1 *very little*–5 *very much*), how much they knew about the topic (1 *no knowledge at all*–5 *great deal of knowledge*), and which of the two positions taken by scientists in the dispute they thought more likely to be correct. Respondents then saw the 30 “possible causes of scientific disagreements” and were asked to rate them on a fully-labeled scale (1 *never a cause of disagreements*, 2 *seldom* …, 3 *sometimes* …, 4 *often* …, 5 *almost always a cause of disagreements*). The survey instrument finished with demographic questions (gender, age, education, science education, political ideology (1 = *strongly conservative*, 5 = *strongly liberal*), and ethnicity).

Participants also responded to several questions originally aimed at views of science education [32]. These variables are not relevant to the current investigation and we do not report the results in this paper. More details are available from the first author upon request.

#### Analysis

We used exploratory principal axis factoring (EFA) with oblique rotation to examine the dimensionality of the candidate Science Dispute Reason items combining data from all three scenarios. Factors with eigenvalues larger than 1 were extracted. Item response theory (IRT) methods were then used to evaluate individual item and overall test functioning for each extracted factor. We used the Graded Response Model (GRM) framework for ordinal response scales as implemented in the ltm package for the R statistical computing environment [33]. Both unconstrained (discrimination parameters allowed to vary) and constrained Rasch (discrimination parameters fixed across items) GRMs were fit and model comparisons were made with the Bayesian Information Criterion (BIC). GRM results were used to reduce the item pool using the following criteria: 1) Individual items should show good item response curves and superior discrimination compared to the other items, 2) the final item set should fit the constrained (Rasch) GRM model to justify simply summing the items to create a total score, 3) the total test information function should be roughly centered on zero, 4) the final items chosen should be face valid and span the conceptual facets of the latent trait, and 5) test items should not show any differential item functioning (DIF) with respect to scenario, education or gender. Average reliability estimates were calculated using IRT person-separation [34] and Cronbach’s alpha.

### Results

#### Exploratory factor analysis

EFA resulted in a 3-factor solution (eigenvalues were 12.71, 2.47 and 1.28, respectively) accounting for 54.86% of the variance in the item set. Table 1 shows factor loadings for each item on each extracted factor after oblimin (oblique) rotation. Several cross-loading items (# 10, 11, 13, 29) were removed before proceeding with the IRT modeling.

**Table 1:** Study 1. Reasons for science disputes principal axis factoring results.

Item	Process/ Competence	Interests/ Values	Complexity/ Uncertainty
**1. Scientists use different measuring tools.**	**0.61**		
**2. Scientists use different research methods.**	**0.80**		
**3. Techniques for analyzing study data differ from study to study.**	**0.68**		
4. Scientists disagree on the best research designs to test their ideas.	0.50		
5. Scientists focus on slightly different variables in their research studies.	0.53		
6. Different studies use different research designs.	0.68		
**7. Scientists vary in competence.**	**0.52**		
**8. Scientists have different credentials for this research.**	**0.60**		
**9. Scientists vary in their experience studying the issue.**	**0.65**		
10. Scientists put different amounts of effort and care into their research.	0.38	0.32	
11. Scientific studies can be done badly.	0.32	0.37	
12. Scientists can miss important information.		0.33	
13. Scientists compete with other scientists.	0.41	0.45	
**14. Scientists conform to what their close colleagues want.**		**0.72**	
**15. Scientists are influenced by the chance for money and promotion.**		**0.78**	
16. Businesses, governments, and activists press scientists to take certain positions.		0.56	
**17. Scientists want recognition and a good reputation.**		**0.55**	
18. Employers persuade their scientist employees to reach certain conclusions.		0.67	
**19. Scientists tend to reach conclusions that fit their personal values.**		**0.73**	
20. Their life experiences affect scientists’ conclusions.		0.45	
**21. Scientists’ views on science are affected by their political beliefs.**		**0.76**	
22. Scientists’ values affect their conclusions about science.		0.70	
23. Scientists’ desire for “elegant solutions” affects their conclusions.		0.58	
**24. Scientists reach conclusions shaped by what they want to believe.**		**0.68**	
**25. The topic is so complex scientists may not realize they’re grasping only part of it.**			**0.45**
**26. The topic is too unpredictable.**			**0.73**
**27. The topic is too complex to get clear results.**			**0.62**
**28. Reaching general conclusions on the topic is hard.**			**0.52**
29. Too many factors influence results for this topic.	0.34		0.43
**30. There is too much uncertainty in this area for definite answers.**			**0.79**
**Cronbach’s Alpha** (final bolded items)	**.86**	**.87**	**.84**
**Average IRT reliability** (final bolded items)	**.86**	**.87**	**.84**

Note: Items selected for final 17-item scale in **bold**. Partial factor loadings < .32 not shown.

#### Graded response models

Constrained (Rasch) and unconstrained graded response models (GRMs) were fit separately to the remaining items for each of the three subscales.

Process/Competence. The constrained (Rasch) model fit the nine remaining items significantly better than an unconstrained model, BICΔ = 27.79, and showed strong item discrimination (discrimination = 2.10 [35]). We reduced the item set one at a time as described above (Analysis) to find a parsimonious subset of items with strong reliability. The final subscale was composed of six items, which fit the Rasch model (discrimination = 2.11) and showed strong reliability (Table 1). None of the items showed substantial DIF across gender, education, or scenario read (all models assuming DIF resulted in less than 2% additional explained variance).

Interest/Values. The unconstrained model fit the 12 bias items significantly better than a constrained (Rasch) model, BICΔ = 34.63, suggesting that some items had higher discrimination than others. We reduced the item set one at a time as described above to find a parsimonious subset of items with strong reliability. The final subscale was composed of six items (Table 1), for which the constrained (Rasch) model fit significantly better than an unconstrained model, BICΔ = 9.22, and showed strong item discrimination (discrimination = 2.23). None of the items showed substantial DIF across gender, education, or scenario read (all models assuming DIF resulted in less than 2% additional explained variance).

Complexity/Uncertainty. The constrained (Rasch) model fit the five remaining complexity items significantly better than an unconstrained model, BICΔ = 9.94, and showed strong item discrimination (discrimination = 2.20). None of the items showed substantial DIF across gender, education, or scenario read (all models assuming DIF resulted in less than 2% additional explained variance).

Correlations among subscale scores (averaging items for each final subscale) were *r* = .68 (Process/Competence & Complexity/Uncertainty), *r* = .59 (Process/Competence and Interests/Values), and *r* = .49 (Interests/Values & Complexity/Uncertainty).

#### Associations between demographics, topic-related perceptions, and Science Dispute Reasons

Table 2 shows measures of location and dispersion for each Science Dispute Reason for each dispute topic. Mean differences of ~0.16 are conventionally significant (*p* < .05) given these sample sizes. Using this criterion, there were no differences in process/competence reasons between topics, and the Dark Matter and Marijuana topics had lower Interest/Values and Complexity reasons ratings, respectively. We also examined the associations between Science Dispute Reasons and demographic (age, gender, education, political ideology) and topic-related (perceived knowledge about topic, care about topic, heard of disputes in the past) variables for each scenario. Education level (high school or less vs some college or vocational vs college or more) was not significantly related to Science Dispute Reasons for any of the topics. For the climate change topic, all correlations were very small (*r*s < .10) with the exception of more liberal participants reporting Process/Competence (*r* = -.11), Interests/Values (*r* = -.22), and Complexity (*r* = -.15) reasons as less likely as compared to more conservative participants. For the marijuana topic, participants who were older (*r* = .18), cared more (*r* = .16), and had heard of disputes in the past (*r* = .22) rated the Process/Competence reasons as more likely. For the dark matter topic, participants who had heard of disputes in the past reported Process/Competence (*r* = .19), Interests/Values (*r* = .15), and Complexity (*r* = .19) reasons as more likely as compared to participants who had not heard of disputes. More liberal participants also reported Interests/Values reasons (*r* = -.15) as less likely as compared to more conservative participants.

**Table 2:** Study 1. Science Dispute Reason descriptive statistics by scenario.

	Climate (*n* = 208)	Marijuana (*n* = 196)	Dark Matter (*n* = 201)
**Process/Competence**			
Mean (sd)	3.51 (0.74)	3.46 (0.70)	3.43 (0.70)
Median (mad)	3.50 (0.74)	3.33 (0.49)	3.33 (0.49)
**Interests/Values**			
Mean (sd)	3.34 (0.85)	3.28 (0.78)	3.09 (0.73)
Median (mad)	3.33 (0.74)	3.33 (0.74)	3.00 (0.74)
**Complexity/Uncertainty**			
Mean (sd)	3.48 (0.83)	3.22 (0.72)	3.45 (0.80)
Median (mad)	3.60 (0.89)	3.20 (0.59)	3.40 (0.59)

### Discussion

The final scale included 17 items on three correlated subscales: Process/Competence (comprising three items each from the original Methods and Competence categories—see Item Generation, above), Interests/Values (comprising three items each from the original Personal Benefits and Values categories), and Complexity/Uncertainty (comprising five items from the original Complexity/Uncertainty category). All subscales were consistent with previous research, particularly that of Johnson & Dieckmann [12] also using a U.S. sample; as before, participants did not distinguish between personal interests and values as reasons for disputes, nor between competence and methods. The research process or methods subscale observed in an educated German sample [13] and for the biology topic in an Israeli student sample [14] was here folded by lay responses into the Competence subscale, but otherwise these factor structures were consistent with the German and Israeli findings. All subscales exhibited good reliability and item discrimination, with no differential item functioning across gender, education, or topic scenario (i.e., item functioning did not differ by the nature of the dispute shown to participants). All associations between Science Dispute Reasons and demographic variables were small, with having heard of disputes in the past and political ideology being the largest effects depending on topic. Political ideology associations related to all Science Dispute Reasons—i.e., exhibited a global effect, as discussed earlier—for the climate change topic (although the association was largest for Interests/Values) but were specific to Interest/Values for the Dark Matter topic. As the main function of Study 1 was to run psychometric analyses to create a Science Dispute Reasons scale for U.S. samples to parallel the ECSC scale created with German subjects [13], it was not designed to test our hypotheses related to explanatory factors. That was the aim of Study 2.

## Study 2

In Study 2, we replicated the psychometric findings from Study 1 and tested our hypotheses regarding potential explanatory factors outlined earlier (see Fig 1). We used two new dispute topics (Salt intake and Nanotechnology) designed to vary along the familiar to emergent/unknown continuum. We expected controversies regarding salt intake to be relatively familiar and mundane to participants, and therefore, less likely to activate worldview-consistent but perhaps still to elicit conspiratorial thinking (e.g., if they expect profit motives to drive some scientists). Several studies have examined public perceptions of nanotechnology and have found that the public tends to have relatively positive attitudes toward nanotechnology even when having relatively little knowledge about the topic and not necessarily trusting government agencies to manage risks [36, 37]. Several reviews of this literature have concluded that public perceptions of nanotechnology might be driven more by trust, general views of science, religiosity, and worldviews [38, 39], implying that political ideology and, potentially, conspiracist ideation, might have stronger relations to Science Dispute Reasons in the nanotechnology than for dietary salt.

### Methods

#### Participants

Members of the Survey Sampling International, Inc. online panel who were Americans 18 or older, but who had not participated in the earlier study, were randomly recruited October 17–20, 2017. The full sample consisted of 413 participants, but a subset of 40 completed the survey so quickly (less than 10 minutes) as to create doubt that they were attending to the content. They were removed and our final sample included 373 participants. They were 54% female, with a mean age of 45.2 years [range 18–84], 20% with a high school degree or less, 46% with a college degree or more, and 81% identifying as non-Hispanic white.

#### Procedure and measures

After answering several demographic questions, participants were asked the dispute awareness, interest and knowledge questions (Study 1, Methods) and randomly assigned to one of two dispute scenario conditions (Salt or Nanotechnology; see Appendix for scenario text). They indicated their level of caring about which side was correct and subjective topical knowledge again as in Study 1. Participants then responded to the Dispute Reasons items, and a series of individual difference measures described below.

Cognitive/numerical skills. Finally, four multiple-choice problems of the Berlin numeracy test ([40]; e.g., *Imagine we are throwing a five-sided die 50 times*. *On average*, *out of these 50 throws how many times would this five-sided die show an odd number (1*, *3 or 5)*?) were followed by nine number series problems intended to measure fluid reasoning, in which the task is to complete the sequence by filling in the blank (e.g., *10*, *4*, *___*, *-8*, *-14*, *-20*; adapted version of number series test used in Dieckmann et al. [21]).

Participants also answered for “the issue described in this paragraph” importance (three items; e.g., 1 *significant*-7 *insignificant*) and relevance (four items; e.g., 1 *meaningful*-7 *meaningless*) semantic differential items from the IRA index of risk communication effects [41]. For parsimony, these measures are not included in the current paper but results are available from the first author upon request.

Science-specific knowledge. A scale probing knowledge about science facts and reasoning included seven true-false items (e.g., “All radioactivity is man-made”) and two items about the existence and length of the Earth’s solar orbit [42]. A second scale probed familiarity with scientific reasoning ([43] as a count of correct answers to 11 true-false items. For example, the item probing understanding of reliability was “A researcher develops a new method for measuring the surface tension of liquids. This method is more consistent than the old method. True or False? The new method must also be more accurate than the old method”).

Science-specific beliefs. Scales probing attitudes and beliefs about science included beliefs about scientific positivism (taken from Steel et al. [44] and Rabinovich & Morton [45]; eight items, e.g., “Science provides objective knowledge about the world,” 1 = *strongly disagree*, 5 = *strongly agree;* Cronbach’s alpha = .77, in this sample) and the general credibility of science and scientists, or mistrust (CoSS scale [26]; six items, e.g., “People trust scientists a lot more than they should,” 1 = *disagree very strongly*, 7 = *agree very strongly;* all items reverse coded so higher scores equal higher credibility of science; Cronbach’s alpha = .88 in this sample).

General attitudes/worldviews. Participants then responded to the 15-item Generic Conspiracist Beliefs scale [46]—e.g., “Groups of scientists manipulate, fabricate, or suppress evidence in order to deceive the public” (1 *definitely not true*, 5 *definitely true*; its factors include government malfeasance, extraterrestrial cover-up, malevolent global conspiracies, personal well-being, and control of information; Cronbach’s alpha = .93 in this sample). Participants also responded to a single item asking about political ideology (1 = *strongly conservative*, 5 = *strongly liberal)*.

#### Analysis

The final scale items retained from Study 1 were evaluated with a confirmatory factor analysis followed by the same IRT and differential item functioning analyses described above. We then examined the relations between the three Dispute Reasons subscales and topic-related, demographic and individual difference variables in a structural equation modeling (SEM) framework. Each Science Reason Dispute subscale was modeled as an endogenous (outcome) variable predicted by the individual difference variables (political ideology, cognitive ability as measured by the number series test, familiarity with scientific reasoning, factual scientific knowledge, credibility of science, belief in positivism, and conspiracist Ideation) and control variables (gender, age, perceived knowledge of domain, care about domain, and heard of scientific disputes in the past). Numeracy and educational status were not substantial or significant predictors in any model and were removed for model parsimony with no substantive effect on other model parameter estimates. Based on previous empirical results relating political ideology and conspiracist ideation to credibility of science (e.g., [26]), we included two mediation paths with political ideology and conspiracist ideation exerting indirect influences on the Dispute Reasons outcomes through credibility of science. The direction of these causal arrows make intuitive and theoretical sense in that political ideology and a tendency toward conspiratorial thinking could impact perceptions of the credibility of science, whereas it is less plausible that perceptions of the credibility of science would directly change one’s political ideology or tendency toward general conspiratorial thinking. Fig 1 shows the conceptual model for each participant group, fit separately for participants randomly assigned to the Salt and Nanotechnology dispute scenarios. Exogenous predictor variables with nonsignificant correlations (*r* < .10) were fixed to 0. There is disagreement in the SEM literature regarding precise cutoffs for model fit statistics. In this study, model fit was assessed by RMSEA (~.06 or less indicating good fit with values >.10 indicating poor fit; [47]), SRMR (~.08 or less indicating good fit; [48]), and CFI (~.90 or higher indicating good fit; [48]). All statistics are reported so that readers may apply any more conservative or liberal criteria as they see fit.

### Results

#### Psychometrics of Science Dispute Reasons scale (CFA & IRT)

A confirmatory factor analysis loading the final Science Dispute items on three correlated factors resulted in “good” fit per the SRMR with the 90% CI for RMSEA below the cutoff for poor fit and the CFI very close to .90 (RMSEA = .08, 90% CI = .07, .09; CFI = .89; SRMR = .05). Overall, the quality of the model fit depended on the fit statistic used but we deemed it good enough overall to proceed to IRT modeling on the subscales. A constrained (Rasch) model fit the Process/Competence (BICΔ = 25.86), Interest/Values (BICΔ = 9.14), and Complexity/Uncertainty (BICΔ = 5.62) subscale items better than an unconstrained model. Cronbach’s alpha reliability (IRT reliability) estimates were .81 (.82), .82 (.81) and .82 (.82) for the Competence/Process, Interest/Values, and Complexity/Uncertainty subscales, respectively. None of the items showed substantial DIF across gender, education, or scenario (all models assuming DIF resulted in less than 4% additional explained variance). Correlations among the subscale scores (averaging items for each subscale) were *r* = .59 (Process/Competence & Complexity/Uncertainty), *r* = .57 (Interests/Values & Complexity/Uncertainty), and *r* = .55 (Process/Competence & Interests/Values). These results replicate Study 1’s findings regarding the psychometric properties of these subscales.

#### Relations between Science Dispute Reasons, topic-related and individual difference variables (SEM)

Table 3 shows descriptive statistics and zero-order correlations for each study variable. Among the largest correlations are positive relations between number series, scientific reasoning and scientific knowledge; negative relations between conspiracist thinking and the same three variables; liberal ideology positively related to credibility of science and negatively to conspiracist thinking; and subjective knowledge of the topic positively related to caring about the topic and belief in scientific positivism.

**Table 3:** Study 2. Pearson correlations for participants who read the Salt (*n* = 201) and Nanotechnology (*n* = 172) scenarios.

	1	2	3	4	5	6	7	8	9	10	11	12	13	14
1. Process /Competent														
2. Interest /Values	**.57** (**.53**)													
3. Complexity /Uncertainty	**.55** (**.63**)	**.61** (**.54**)												
4. Number Series	-.01 (.10)	-.06 (.01)	-.15 (.14)											
5. Science reasoning	.02 (.11)	.06 (**.21**)	-.08 (.17)	**.44** (**.25**)										
6. Science knowledge	-.04 (.07)	-.04 (.14)	**-.26** (**.20**)	**.40** (**.38**)	**.45** (**.42**)									
7. Positivism	.05 (-.01)	.03 (-.14)	.10 (-.09)	-.15 (-.05)	**-.21** (**-.25**)	-.11 (**-.21**)								
8. Credibility of Science	-.08 (-**.23**)	**-.22** (-**.27**)	**-.20** (-**.20**)	.17 (.15)	.08 (.12)	**.21** (**.21**)	-.08 (.07)							
9. Political Ideology	-.04 (.01)	-.15 **(-.20**)	-.15 (-.06)	.09 (.02)	-.12 (-.09)	.07 (-.14)	.15 (.08)	**.36** (**.40**)						
10. Conspiracist ideation	.05 (.02)	.08 (.11)	**.22** (.02)	**-.40** (**-.23**)	**-.31** (**-.40**)	**-.36** **(-.34)**	**.31** (**.21**)	**-.42** (-**.33**)	-.09 (.09)					
11. Age	.10 (.19)	.09 (.04)	-.01 (.09)	.13 (-.07)	**.26** (**.13**)	**.28** (-.01)	**-.23** (-.15)	.03 (.01)	-.09 (-.08)	**-.23** (**-.18**)				
12. Care about topic	.15 (.07)	.09 (.05)	.11 (.02)	-.06 (-.09)	-.09 (-.01)	-.10 (-.05)	**.31** (.19)	-.04 (.09)	.08 (.13)	.16 (.07)	.06 (.14)			
13. Know of topic	.08 (**-.26**)	.11 (-.03)	.06 (-.15)	-.13 (-.11)	-.11 (**-.20**)	-.06 (**-.20**)	**.38** (**.37**)	**-.27** (-.09)	-.02 (-.06)	**.25** (**.33**)	.03 (-.17)	**.41** (**.37**)		
14. Dispute aware	.14 (**.15**)	.11 (**.25**)	.01 (.15)	**.22** (.15)	.11 (.14)	**.29** (**.20**)	.12 (.07)	-.10 (.01)	-.05 (-.06)	-.15 (-.07)	.07 (.03)	.13 (**.20**)	.16 (**.33**)	
*Mean*	3.42 (3.39)	3.37 (3.32)	3.28 (3.40)	3.09 (3.26)	5.55 (5.70)	7.20 (7.24)	3.42 (3.45)	4.13 (4.16)	2.88 (2.78)	2.19 (2.16)	45.6 (44.7)	3.78 (3.60)	3.13 (2.33)	- -
*Median*	3.33 (3.33)	3.33 (3.33)	3.20 (3.40)	3.00 (3.00)	5.00 (6.00)	7.00 (7.00)	3.38 (3.38)	4.00 (4.25)	3.00 (3.00)	2.07 (2.13)	42.0 (39.5)	4.00 (4.00)	3.00 (2.00)	- -
*SD*	.69 (.66)	.77 (.75)	.73 (.79)	2.68 (2.62)	2.00 (2.03)	1.42 (1.44)	0.67 (0.66)	1.22 (1.28)	1.15 (1.25)	0.71 (0.69)	18.9 (19.7)	1.03 (1.17)	0.97 (1.21)	- -

*Note*. Values *not* in parentheses are for the salt the scenario. Values in parentheses are for the nanotechnology scenario. Values in the correlation matrix are Pearson correlations with effects at *r* ≥ .20 bolded. The dispute aware variable (14) is binary so the mean, median, and SD are not reported.

Table 4 shows the SEM results for participants who were randomly assigned to the Salt dispute scenario (*N* = 201). The final model resulted in “good” fit based on the SRMR, with the RMSEA very close to .06 (i.e., close to “good” and far from “poor” based on conventional criteria), and the CFI very close to .90 (RMSEA = .07, 90% CI = .05, .10; CFI = .87; SRMR = .06). There were relatively few significant direct effects on Science Dispute Reasons for the Salt scenario. Participants who had heard of scientific disputes in the past reported that competence/methods reasons were more likely as compared to those who had not heard of scientific disputes. Participants with higher perceived credibility of science (lower mistrust) reported lower likelihood ratings for the Interests/Values reasons, and participants higher in scientific knowledge reported lower likelihood ratings for the Complexity/Uncertainty reasons for this Salt dispute. As expected, there were also significant direct effects of political ideology (more liberal ideology -> higher credibility of science) and conspiracist ideation (higher conspiracist ideation -> lower credibility of science) on credibility of science. Both political ideology and conspiracist ideation had significant indirect effects on the Interests/Values reasons through credibility of science, such that more conservative ideology and higher conspiracist ideation related to higher perceived likelihood of Interests/Values causes mediated through decreases in perceived credibility of science. Overall, variance explained was modest for the Science Dispute Reasons scores (6%-14%) with a relatively large amount of variability in the credibility of science scores (26%) explained by political ideology and conspiracist ideation.

**Table 4:** Study 2. Direct/Indirect effects from SEM for participants who read the Salt scenario (*N* = 201).

	Process/Comp	Interest/Values	Complex/Unc	Cred of Science
**Direct effects**				
Fluid intelligence (number series)	0.01 (-0.16, 0.17)	-0.07 (-0.23, 0.09)	-0.04 (-0.20, 0.12)	
Science reasoning (SRS)	0.04 (-0.13, 0.21)	0.12 (-0.05, 0.28)	0.06 (-0.11, 0.22)	—
Science factual knowledge	-0.08 (-0.26, 0.10)	-0.09 (-0.27, 0.09)	**-0.24** **(-0.41, -0.07)**	—
Belief in positivism	0.01 (-0.16, 0.16)	0.01 (-0.15, 0.16)	0.08 (-0.08, 0.23)	—
Credibility of science	-0.08 (-0.24, 0.09)	-0.19 (-0.35, 0.03)	-0.10 (-0.26, 0.06)	**—**
Political ideology	0.01 (-0.14, 0.17)	-0.04 (-0.19, 0.11)	0.09 (-0.24, 0.05)	**0.32** **(0.21, 0.44)**
Conspiracist ideation	0.04 (-0.13, 0.22)	0.01 (-0.16, 0.18)	0.10 (-0.07, 0.27)	**-0.39** **(-0.50, -0.28)**
Age	0.11 (-0.04, 0.25)	0.09 (-0.06, 0.24)	0.05 (-0.09, 0.20)	—
Care about topic	0.12 (-0.03, 0.27)	0.04 (-0.12, 0.19)	0.08 (-0.07, 0.23)	—
Know about topic	-0.03 (-0.19, 0.13)	0.01 (-0.15, 0.18)	-0.09 (-0.25, 0.07)	**—**
Heard of disputes	**0.15** **(0.01, 0.30)**	0.12 (-0.02, 0.27)	0.08 (-0.06, 0.23)	**—**
**Indirect effects**	**Estimate**			
Conspir -> Cred -> Competence	0.03 (-0.03, 0.09)			
Ideo -> Cred -> Competence	-0.03 (-0.08, 0.03)			
Conspir -> Cred -> Ints/Vals	**0.07** **(0.01, 0.14)**			
Ideo -> Cred -> Ints/Vals	**-0.06** **(-0.12, -0.01)**			
Conspir -> Cred -> Complexity	0.04 (-0.02, 0.10)			
Ideo -> Cred -> Complexity	-0.03 (-0.08, 0.02)			
**Variance Explained (R**^**2**^**)**	6%	9%	14%	26%

Note: Cell entries are standardized regression coefficients (95% CIs). Estimates with 95% CIs that do not include 0 are bolded. These estimates are traditionally significant at p < .05.

Table 5 shows the SEM results for participants who were randomly assigned to the Nanotechnology dispute scenario (*N* = 172). The final model resulted in “good” fit per the SRMR with the RMSEA very close to .06 and the CFI very close to .90 (RMSEA = .08, 90% CI = .05, .10; CFI = .88; SRMR = .06). There were many more significant direct effects and much more variance explained for the Nanotechnology scenario as compared to the Salt scenario. Participants with higher credibility of science scores, higher self-reported knowledge about nanotechnology, and those who had not heard of disputes in the past reported lower likelihood ratings for all three Science Dispute Reasons. Higher positivism and care scores were related to higher likelihood ratings for the Process/Competence reason. Higher scientific reasoning and conspiracist ideation scores were related to higher likelihood ratings for the Interests/Values reason. Higher science knowledge was related to higher likelihood ratings for the Complexity/Uncertainty reason, an effect opposite to that in the Salt scenario. As with the Salt scenario, there were also significant direct effects of political ideology (more liberal ideology -> higher credibility of science) and conspiracist ideation (higher conspiracist ideation -> lower credibility of science) on credibility of science. Both political ideology and conspiracist ideation had significant indirect effects on all three Dispute Reasons through credibility of science, such that more conservative ideology and higher conspiracist ideation related to higher perceived likelihood of all reasons mediated through decreases in perceived credibility of science. Overall, a substantial amount of variability in the Science Dispute Reasons scores was explained by the predictor set (19%–29%) with almost one third of variability in the credibility of science scores (31%) explained by political ideology and conspiracist ideation.

**Table 5:** Study 2. Direct/Indirect effects from SEM for participants who read the Nanotechnology scenario (*N* = 172).

	Process/Comp	Interest/Values	Complex/Unc	Cred of Science
**Direct effects**				
Fluid intelligence (number series)	0.08 (-0.07, 0.22)	-0.05 (-0.20, 0.10)	0.08 (-0.07, 0.23)	
Science reasoning (SRS)	0.06 (-0.10, 0.21)	**0.20** **(0.05, 0.35)**	0.10 (-0.06, 0.26)	—
Science factual knowledge	0.05 (-0.13, 0.23)	0.09 (-0.09, 0.26)	**0.18** **(0.00, 0.36)**	—
Belief in positivism	**0.15** **(0.01, 0.29)**	-0.04 (-0.19, 0.11)	0.03 (-0.12, 0.19)	—
Credibility of science	**-0.31** **(-0.47, -0.16)**	**-0.21** **(-0.37, -0.05)**	**-0.28** **(-0.44, -0.11)**	**—**
Political ideology	0.10 (-0.06, 0.24)	-0.12 (-0.27, 0.03)	0.05 (-0.11, 0.21)	**0.42** **(0.31, 0.54)**
Conspiracist ideation	0.10 (-0.06, 0.26)	**0.23** **(0.07, 0.39)**	0.13 (-0.04, 0.30)	**-0.36** **(-0.48, -0.24)**
Age	0.13 (-0.01, 0.27)	-0.01 (-0.15, 0.13)	0.07 (-0.07, 0.22)	—
Care about topic	**0.15** **(0.01, 0.30)**	0.09 (-0.06, 0.24)	0.08 (-0.07, 0.24)	—
Know about topic	**-0.43** **(-0.58, -0.27)**	**-0.17** **(-0.34, -0.01)**	**-0.23** **(-0.40, -0.06)**	**—**
Heard of disputes	**0.22** **(0.08, 0.36)**	**0.26** **(0.12, 0.41)**	**0.16** **(0.01, 0.31)**	**—**
**Indirect effects**	**Estimate**			
Conspir -> Cred -> Competence	**0.11** **(0.04, 0.18)**			
Ideo -> Cred -> Competence	**-0.13** **(-0.21, -0.06)**			
Conspir -> Cred -> Ints/Vals	**0.08** **(0.01, 0.14)**			
Ideo -> Cred -> Ints/Vals	**-0.09** **(-0.16, -0.02)**			
Conspir -> Cred -> Complexity	**0.10** **(0.03, 0.17)**			
Ideo -> Cred -> Complexity	**-0.12** **(-0.19, -0.04)**			
**Variance Explained (R**^**2**^**)**	29%	25%	19%	31%

Note: Cell entries are standardized regression coefficients (95% CIs). Estimates with 95% CIs that do not include 0 are bolded. These estimates are traditionally significant at p < .05.

### Discussion

Study 2 confirmed the psychometric properties of three major groups of reasons used by the U.S. public to explain scientific disputes. It also identified several individual differences that influenced the likelihood of Science Dispute Reasons depending on the scientific dispute in question. Our first hypothesis that cognitive ability and science-specific knowledge would be positively associated with complexity reason ratings received partial support. Cognitive ability was not a unique predictor of Science Dispute Reason ratings and knowledge of science reasoning was not related to Complexity/Uncertainty reason ratings. However, higher familiarity with science reasoning was related to higher ratings of Interests/Values reasons from the nanotechnology topic, suggesting that those with a better understanding of how science works recognize the influence of these reasons. Knowledge of science facts was related to Complexity/Uncertainty reason ratings for both topics, albeit in opposite ways (higher science knowledge related to lower/higher Complexity/Uncertainty ratings in the salt and nanotechnology topics, respectively). As for science-specific beliefs, we replicated the global credibility of science effects observed in prior work but only for the nanotechnology topic. Belief in scientific positivism did not have the global effects observed in prior studies, only showing a direct positive relation to Process/Competence ratings for the nanotechnology but not salt topics. Finally, on general attitudes/worldviews our second hypothesis that political ideology will have direct and indirect effects that are global, or specific to Interests/Values and Process/Competence, also received partial support. Political ideology lacked any unique direct effects on Science Dispute Reasons, but showed a unique indirect effect on Interest/Values reasons through decreased credibility of science for the Salt topic. For the nanotechnology topic, there was a global indirect effect with more conservative participants rating all Science Dispute Reasons higher. Our third hypothesis that conspiracist ideation will have specific direct and indirect effects on Interest/Values reasons also received partial support. For the salt topic, conspiracist ideation lacked unique direct effects on Science Dispute Reasons but showed a unique indirect effect on Interest/Values reasons through decreased credibility of science for the Salt topic. For the nanotechnology topic, there was a specific direct effect from conspiracist ideation to Interest/Values reasons. This effect is somewhat counterintuitive given the small zero-order correlations between these variables and is evidence of suppressor relation [49, 50]. Further tests identified knowledge of both science fact and scientific reasoning as the variables likely responsible for suppressing the zero-order relation between conspiracist ideation and Interests/Values reason ratings (i.e., both variables are negatively correlated with conspiracist ideation but positively correlated with Interests/Values ratings). Thus, our inclusion of these two knowledge measures in the model allowed us to uncover the relation between these variables that would otherwise have been suppressed. There were also global indirect effects of conspiracist ideation on all Dispute Reason ratings through decreases in credibility of science.

## General discussion

### Measuring science dispute reasons

Following in the footsteps of previous work [12, 13], we developed a Science Dispute Reasons scale that was psychometrically stable and discriminating across five intra-science dispute contexts varying widely in familiarity and salience: the nature of dark matter in the universe; the risks and benefits of marijuana and nanotechnology, respectively; the degree of sea level rise by the end of this century; and how much dietary salt intake should be reduced. Furthermore, this new scale was consistent with the Johnson & Dieckmann [12] factor analyses, both finding in samples of the general U.S. public three different classes of reasons laypeople offer for scientific disputes. Within these classes there is no discrimination between competence and process/method reasons, no discrimination between personal benefit and values (unconscious) reasons, but with topic complexity/uncertainty separated. The German studies found a distinction between competence and process reasons, but otherwise yielded a similar taxonomy (they did not include values measures). Whether this lone difference is due to methodological or sample differences, or to something else, remains to be seen. The Israeli study [14] found a narrower set of reasons applied in different ways across two science disciplines. However, the overall convergence in lay reasons across these few initial studies enhances confidence in the robustness of these findings.

These three classes of reasons offer plausibly distinct explanations: disputing scientists include one group that is more competent than the other, one or both groups’ scientific work is influenced by values or self-interest, or the topic is too complex and uncertain for scientists to (currently) converge on an answer. These reasons do not cover all possibilities explaining scientific disputes, as social constructionist and other arguments in the sociology and philosophy of science have shown over the years [51, 52]. However, those same literatures show these causes are certainly active in scientific work. Furthermore, if laypeople were to try to use these explanations to decide which side in a scientific dispute was more likely to be correct, they might have evidence on which to judge (most for bias, probably least for competence [53, 54]).

We also note that the process/competence and interests/values conflations by Americans in [12] and this study, despite these studies using different analytic approaches, offer intriguing bases for further exploration. For example, perhaps Americans in general—or less science-familiar respondents—conflate choice of methods (process) with competence because they cannot conceive of multiple methods being competent: i.e., there is only one correct way to study a given scientific problem. Perhaps the German sample in [13] did not exhibit this conflation because the German educational system overall, or at the high levels of education earned by this sample, had been exposed to more or different information about this aspect of scientific process.

### Predictors of science dispute reasons

These results also provide some insight as to why one reason might be favored over another. We find a mixed pattern of global and specific impacts on reason likelihood ratings. Both political ideology and conspiracist ideation showed global effects (mediated through credibility of science) on the likelihood ratings of all Science Dispute Reasons. This suggests that laypeople who see science as not credible are not exclusively making reason judgments systematically, as considered judgments one reason at a time. If they were, we would expect to see specific ratings patterns exclusively. For instance, bias would be rated relatively high as a factor clearly making scientists untrustworthy, particularly for those who also favor conspiracist ideas. Competence might be rated secondary as even if all scientists are deemed incompetent this reason posits some as more competent than others, making them perhaps a bit less untrustworthy. And complexity would be rated last as a reason unrelated to qualities of scientists themselves and thus to credibility. These global effects, regardless of the type of reason, imply that this is more a signal of general mistrust—“knowing what I do about scientists, *any* of these explanations for their disagreements could be valid”—than an explanation of reason choice.

We also find global reason effects for topic-related perceptions (i.e., knowledge of dispute domain, care about dispute domain, heard of disputes) for the more unfamiliar nanotechnology topic but not dietary salt. This suggests that these perceptions might have larger impacts for more unfamiliar dispute topics. However, we also see a pattern of specific reason effects that suggest more nuanced and systematic consideration of Science Dispute Reasons based on both worldview orientation (political ideology and conspiracist ideation related to Interest/Values reason ratings) and knowledge about science (related to Complexity reason ratings). Study 1’s findings that dark matter prompted less attribution of the dispute to interests/values, but marijuana less attribution to complexity reasons, also suggest that laypeople were making plausible inferences about the reason given the nature of the topic and dispute (e.g., how could scientists make money off equally hypothetical particles?). Our observed topic-related differences converge with prior research suggesting that people are sensitive to perceived differences between topics or domains (e.g., [14]) and that there are important interactions between topic characteristics and person qualities (e.g., worldview orientations having larger effects on reason perceptions for unfamiliar or more hotly debated topics).

These findings extend but also diverge from prior research in this area [10, 11, 12]. As discussed in the Background section, high education has been found to foster self-interest [10], or bias and complexity attributions for scientific disputes [11], while lower educational levels foster complexity [10] or competence [11] attribution. However, Johnson and Dieckmann [12] and the present findings included a wider range of cognitive ability, science knowledge/reasoning, and worldview variables and did not find a unique impact of education. We suspect that education was acting as a proxy for more specific cognitive ability or science specific knowledge or reasoning variables omitted in most prior studies. Prior work has also found that higher cognitive ability was related to higher ratings of bias and complexity attributions, and lower ratings of competence [11], while we found no unique effects of cognitive ability after controlling for other relevant variables. However, the earlier [11] study examined a diverse set of topics across seven domains without other variables related to science-specific knowledge and reasoning, making direct comparisons of these findings difficult. The present findings are consistent with prior work [12] with respect to the importance of general perceptions of the credibility of science, but extend that work in showing both global and specific impacts, and that credibility of science perceptions may mediate how worldview variables affect Science Dispute Reasons.

Prior and current results relating to belief in scientific positivism are the most difficult to parse. Prior work suggested that belief in scientific positivism enhanced *all* reasons’ ratings [12], while we found only higher likelihood ratings for competence reasons and only for the nanotechnology topic. As an attempt to measure belief that science identifies objective truth about the universe, the association with higher competence-reason scores seems the most plausible, as competent scientists should not be disagreeing about the truth by (this positivist) definition. These inconsistent results raise the following issues for consideration in future research: 1) the belief-in-positivism items, although psychometrically reliable, are measuring something different than we think, warranting separate qualitative and quantitative assessment of this specific belief; and/or 2) there is something about the relations of belief in scientific positivism and other constructs in these studies that we do not yet grasp, also warranting further qualitative and quantitative research. We believe recent work by Thomm et al. [14] provides a promising path toward understanding how individual epistemic perspectives might directly impact Science Dispute Reasons and interact with other topic-related and individual difference variables.

### Implications

Despite these challenges, these findings carry important implications for understanding lay reactions to scientific disputes and for communicating scientific results under dispute. In general, when laypeople see conflicting scientific claims without any cues as to why this dispute has occurred (as was the case in the scenarios used in this and prior studies), worldview orientations and perceptions of the general credibility of science seem to be the strongest drivers of perceived reasons for the dispute, over cognitive/knowledge factors. This result is not surprising given the relatively large literature on motivated cognition, in that people will tend to seek out information and make judgments and decisions that are consistent with what they want to believe [17, 18, 19]. Furthermore, these drivers may push some members of the public to make dispute attributions that can further undermine trust and support for science (e.g., if laypeople assume disputes are due to scientist self-interest). For instance, we do not know exactly how much these reason inferences affect other important science-related outcomes, such as whether a specific scientific conclusion will be dismissed because of the perceived causes of a dispute. It is unclear to what extent laypeople use other topic/context-specific cues to make reason judgments as opposed to using worldview orientation, for example. We also do not know their relative effect sizes, nor how sensitive these perceptions are to different topics or dispute contexts.

One implication for science communication is that motivated reasoning might be reduced, if perhaps not eliminated, if the causes of scientific disputes were made explicit to the extent feasible. This is only possible if the causes are known in a particular case and this information could be supplied within the communication context. A significant challenge, of course, is that the public can receive conflicting scientific information from many sources and there would be no way to tag all of this source information with all possible causes of conflict. Even more challenging are questions concerning whether mass media or social media sources would publish such information (e.g., see Friedman et al. [55] on the difficulty in getting mass media to discuss scientific uncertainty) and how would we identify who would be a credible source of information on reasons for the dispute. For example, scientists on one or both sides of a dispute could put forward claims that they are collectively more competent and/or more disinterested than their opponents, with hopes of attracting public support. No one to our knowledge has yet collected data on the degree to which this is already occurring, or which factors might influence it. A more general science-communication implication is that greater explicit discussion of why scientists disagree might be warranted. What makes a scientific topic “complex,” for example, and what cues might make this clear to laypeople? Given the challenges for scientists themselves interpreting a given dispute correctly, chances for laypeople with less scientific expertise may be minimal [53]. However, there are emerging resources for helping them interpret scientific claims (e.g., [56, 57, 58]) might try to address such questions.

### Limitations

The current study has several limitations that may have introduced bias and/or decreased the generalizability of these results. First, there is always the possibility of unmeasured confounding in the analysis of Study 2 data, so that introducing a range of unmeasured predictors/meditators would alter the observed effects and subsequent conclusions. Second, the observed suppression of the zero-order relation between conspiracist ideation and Interest/Values reasons suggests relatively complex relations between our model predictors. Although the presence of a suppression effect does not always represent a model specification error, it certainly points to the possibility of a more complex theoretical model that describes these variable relations that should be tested and replicated in future work. Third, generalizability of the results is limited by only (non-representative) sampling lay adults from the U.S. and the relatively small number of scientific dispute topics studied.

### Conclusions

Although still a relatively new topic of study, we have made considerable progress in understanding lay perceptions of why scientific disputes occur. First, a measure for assessing perceived reasons for such disputes between groups of scientists has been produced that is both highly reliable and replicable across U.S. samples. Second, we have preliminary evidence that these attributions are more strongly driven by attitudes and worldviews as opposed to objective knowledge and skills. Third, we are beginning to get enough traction to start incorporating some of the larger issues into future research efforts. For example, are perceptions shaped more by attributes of persons or of situations? How might alternative explanations affect scientific authority, scientific advice-following, and other potential consequences? How can we improve science communication in light of these findings? We are excited by the prospects, and encourage other scholars to join in broadening our understanding of this aspect of science and society.

## Appendix

### Study 1

About 85% of all matter in the universe is ‘dark matter’ which scientists know is there due to its gravitational pull on visible matter such as galaxies and radiation, but they cannot see it and do not know what it’s made of. Some scientists think dark matter is made of axions, (currently hypothetical) subatomic particles formed in the core of a star when X-rays scatter off protons and electrons in a strong electric field. Other scientists think dark matter is made of the lightest of the neutralinos, a different set of (currently hypothetical) subatomic particles resulting from the decay of squarks and other relatively heavy particles.

Studies in health damage from drugs disagree about marijuana’s risks and benefits. Some scientists think marijuana produces harmful carcinogens when smoked, is addictive, is a “gateway” drug for more harmful substances, can cause permanent deficits in cognitive function if used by children and young adults, and has fewer medical benefits than people claim. Other scientists think marijuana is significantly less harmful than other legal substances like alcohol and tobacco; evidence on cancer or harder drug use is inconclusive; and marijuana’s illegal federal status has limited full tests of whether it can help those suffering from diseases such as epilepsy, cancer, and glaucoma.

Scientists agree that climate change is occurring with many likely negative effects, but disagree about the magnitude of its potential impacts if current trends continue. For example, some scientists think that by 2100 the average global rise in sea level will be about 1.7 to 3.2 feet, due to both thermal expansion—water expands when it warms—and melting of smaller glaciers, but without large melting of Antarctica, which they think will not happen until much later. Other scientists think Antarctica is closer to substantial melting, and that by 2100 the average global rise in sea level will be about 3.4 to 6.3 feet.

### Study 2

Scientific studies on nutrition agree that Americans eat too much salt, both sprinkled on food and far larger amounts in processed foods (bread, cereal, salad dressing, canned vegetables and soup, ketchup, etc.). But they disagree on what level is safe, especially for people at risk for heart disease and stroke: over half the U.S. population, including those with high blood pressure, older than 51, African Americans, and with diabetes, chronic kidney disease, and congestive heart failure. Some scientists think everyone should reduce intake by one-third, with people at risk cutting salt intake by half. Other scientists think there is little or no health benefit from cutting intake in half or giving different recommendations for at-risk subgroups and others.

Scientific studies on nanotechnology, a new field which exploits how materials change when very small (1,000–8,000 times narrower than a human hair), disagree about its impact. Some scientists stress benefits: medicines (nano-particles can breach the brain-blood barrier, carrying medicine to treat Alzheimer’s or brain cancers hard or impossible to treat now), self-cleaning windows, packaging extending vegetables’ shelf life, pollution cleaners, clothing that repels odors and lasts longer, and so forth. Other scientists stress that the same qualities could impose harm (nano-particles can reach places in the human body larger materials cannot), far more research is done on product development than potential risks, and many benefits claims are so far unproven.
